# Genome Scan for Variable Genes Involved in Environmental Adaptations of Nubian Ibex

**DOI:** 10.1007/s00239-021-10015-3

**Published:** 2021-06-17

**Authors:** Vivien J. Chebii, Emmanuel A. Mpolya, Samuel O. Oyola, Antoinette Kotze, Jean-Baka Domelevo Entfellner, J. Musembi Mutuku

**Affiliations:** 1 School of Life Science and Bioengineering, Nelson Mandela Africa Institution of Science and Technology, P.O. Box 447, Arusha, Tanzania; 2 International Livestock Research Institute (ILRI), Nairobi, Kenya; 3 South African National Biodiversity Institute, Pretoria, South Africa; 4 Biosciences Eastern and Central Africa - International Livestock Research Institute (BecA-ILRI) Hub, Nairobi, Kenya; 5 Central and West African Virus Epidemiology (WAVE), Pôle Scientifique et d’Innovation de Bingerville, Université Félix Houphouët-Boigny, Abidjan, Côte d’Ivoire; 6 Department of Genetics, University of the Free State, Bloemfontein, South Africa

**Keywords:** Nubian ibex, Copy number variation, Genome adaptations, Desert adaptation

## Abstract

The Nubian ibex (*Capra nubiana*) is a wild goat species that inhabits the Sahara and Arabian deserts and is adapted to extreme ambient temperatures, intense solar radiation, and scarcity of food and water resources. To investigate desert adaptation, we explored the possible role of copy number variations (CNVs) in the evolution of Capra species with a specific focus on the environment of *Capra nubiana*. CNVs are structural genomic variations that have been implicated in phenotypic differences between species and could play a role in species adaptation. CNVs were inferred from *Capra nubiana* sequence data relative to the domestic goat reference genome using read-depth approach. We identified 191 CNVs overlapping with protein-coding genes mainly involved in biological processes such as innate immune response, xenobiotic metabolisms, and energy metabolisms. We found copy number variable genes involved in defense response to viral infections (Cluster of Differentiation 48, UL16 binding protein 3, Natural Killer Group 2D ligand 1-like, and Interferon-induced transmembrane protein 3), possibly suggesting their roles in Nubian ibex adaptations to viral infections. Additionally, we found copy number variable xenobiotic metabolism genes (carboxylesterase 1, Cytochrome P450 2D6, Glutathione S-transferase Mu 4, and UDP Glucuronosyltransferase-2B7), which are probably an adaptation of Nubian ibex to desert diets that are rich in plant secondary metabolites. Collectively, this study's results advance our understanding of CNVs and their possible roles in the adaptation of Nubian ibex to its environment. The copy number variable genes identified in Nubian ibex could be considered as subjects for further functional characterizations.

**Supplementary Information:**

The online version contains supplementary material available at 10.1007/s00239-021-10015-3.

## Introduction

The Nubian ibex *(Capra nubiana)* is one of the ten species belonging to the genus Capra*.* The other species are *C. caucasica*, *C. cylindricornis*, *C. falconeri, C. pyrenaica, C. ibex, C. sibirica*, *C. walie*, *C. aegagrus*, and the domestic goat species (*C. hircus*). Nubian ibex is found as small fragmented populations in Egypt, Sudan, Eritrea, Jordan, Oman, Yemen, and Saudi Arabia (Ross et al. 2020). The International Union for Conservation of Nature (IUCN) Red List classifies the Nubian ibex as Vulnerable (Ross et al. 2020). The Nubian ibex is well adapted to hot desert environments characterized by high diurnal temperatures and scarcity of feed and water (Habibi 1994). The Nubian ibex has evolved mechanisms to survive in harsh environments; for instance, it feeds on desert plants rich in secondary metabolites such as alkaloids, suggesting that they have an excellent detoxification system (Hakham and Ritte 1993). Therefore, Nubian ibex’s ability to survive in harsh environments makes it a potential model species for studying environmentally resilient genetic adaptations applicable in other livestock species of economic value.

Copy number variations (CNVs) are genomic structural variants involving duplications or deletions of segments greater than 1000 bp leading to copy number differences among individuals within or between species (Redon et al. 2006). CNVs confer phenotypic effects by changing gene dosage, transcript structure, or regulating genes' expression and functions (Bickhart et al. 2012). Whole gene CNVs affect gene expression such that the higher the number of copies of a gene, the higher its expression levels and vice versa (Butler et al. 2011; Cardoso-Moreira et al. 2016). Exonic CNVs, on the other hand, provide an alternative way of meeting functional demands of a cell through mutually exclusive splicing of the resulting exon duplicates; this allows a single gene to encode functionally diverse proteins (Jones et al. 2012; Kondrashov and Koonin 2001; Letunic et al. 2002). CNVs overlapping with intronic regions of protein-coding genes are likely to affect gene expression since they host regulatory elements such as enhancers, silencers, and non-coding RNAs that modulate transcription (Chorev and Carmel 2012; Rigau et al. 2019).

There is growing evidence of the association between CNVs and a diverse range of phenotypic deviations among species (Bhanuprakash et al. 2018; Iskow et al. 2012); for example, the human genome contains an average of 272 copies of the DUF1220 domain, which is suggested to contribute to the differences in the brain size and cognitive ability between humans and other primates with fewer copies (35 in macaques, 99 in gorillas, and 125 in chimpanzees) (O’Bleness, 2012). Similarly, CNVs of neural development genes (CAST family member 2, Gamma-aminobutyric acid receptor subunit beta-2 and Neuronal PAS domain protein 3) in the domestic goat (Dong et al. 2015), and the behavioral genes (Glutamate Ionotropic Receptor NMDA Type Subunit 2D, Netrin 5 and Neurotrophin-4) in domestic yak (Zhang et al. 2016) may have played a role in the domestication of these species from their respective wild ancestors. A desert-adapted Bactrian camel has two copies each of *NR3C2* and *IRS1* genes known to be involved in water and salt homeostasis, suggesting their role in the desert adaptation of camels compared to their closely related temperate-adapted alpaca (Wu et al. 2014).

Advances in genome sequencing technologies, particularly the whole genome shotgun sequencing platforms such as the Illumina Hiseq 2500, provide approaches such as read-depth, split-read, and paired-end mapping (Alkan et al. 2011) for detecting CNVs from sequence data de novo. Among the sequence-based techniques for calling CNVs, read-depth algorithms can detect exact copy numbers and CNVs in complex genomic regions; hence it is most commonly used for CNV identifications (Alkan et al. 2011). The read-depth approach assumes that the number of reads from a randomly generated shotgun sequence data set that transverse any genomic loci in a reference genome sequence is proportional to the number of copies of the orthologous loci in the test genome. Therefore, gain of copy number events at a given genomic interval in the test genome is seen as higher than average stacking of sequence reads at the orthologous interval in the reference genome, and conversely, a loss of copy number events manifests as a paucity of reads spanning the interval (Abyzov et al. 2011). The recent completion of the domestic goat reference genome (Bickhart et al. 2017) allows in-depth study of adaptive evolution in *Capra* species through CNV screening. This study's objective was to determine CNVs in the Nubian ibex genome compared with the domesticated goat and their potential role in the adaptations to desert habitats.

## Materials and Methods

### Whole Genome Sequence Data

Sequence data for three Nubian ibex individuals were downloaded from the National Center for Biotechnology Information database (https://www.ncbi.nlm.nih.gov/). The data are comprised of sequence data of Nubian ibex individuals obtained from South Africa (Accession number: SRR12990712), Egypt (Accession number: SRR8437789), and Saudi Arabia (Accession number: SRR8437792). The latest reference genome sequence (ARS1 assembly; GCA_001704415.1) (Bickhart et al. 2017) of *Capra hircus* (domestic goat) was downloaded from Ensembl (ftp://ftp.ensembl.org/pub/release-102/fasta/capra_hircus/dna/).

The sequence data downloaded from the public database were assessed for quality using fastQC (Andrews 2010). Adapter sequences in Saudi Arabia data were trimmed using the ILLUMINACLIP function in Trimmomatic version 0.38 (Bolger et al. 2014).

### Mapping and Copy Number Variants (CNV) Calling

Sequence reads for each of the Nubian ibex individuals were aligned to the domestic goat reference genome using Burrow's Wheeler Alignment Maximal Exact Match algorithm (BWA-MEM) version 0.7.15a with parameters set to default (Li and Durbin 2010). Polymerase Chain Reaction (PCR) duplicates were removed using *rmdup* command line of SAMtools (v0.1.8) (Li et al. 2009). The Sequence Alignment Map (SAM) files were converted into Binary Alignment Map (BAM) files using SAMtools 1.4.1(Li et al. 2009).

Copy number variants in each of the Nubian ibex individual genomes were inferred using a read-depth approach implemented in CNVnator (version 0.3.3) (Abyzov et al. 2011), relative to the domestic goat reference genome. CNVnator was set to call CNVs using a genomic window size of 100 base pairs from sequence data for Nubian ibexes sampled from Egypt and South Africa. A genomic window size of 200 base pairs was used to infer CNVs from the Nubian ibex sequence data sampled from Saudi Arabia. The genomic window size for each sequence data was selected based on the authors' recommendations (the ratio of average read-depth signal to its standard deviation should be between 4 and 5) (Abyzov et al. 2011). Other parameters were set to default. The *-unique* flag was used to obtain the zero-mapping quality (q0) score of the calls as per the author's recommendation. The zero-mapping quality (q0) score refers to the fraction of reads in an interval that aligns to more than two locations in the genome.

The output from CNVnator was filtered to retain high quality CNV calls using the following criteria: a call with normalized read depth < 0.7, *p* value < 0.05, q0 > 0.7 was considered a gain of copy number event in the domestic goat reference genome. Similarly, CNV with normalized read depth > 1.20, *p* value < 0.05, q0 > 0.7 was considered a gain of copy number event in the reference genome; with Nubian ibex having more than one copy but fewer than those in the reference. CNV region with normalized read depth > 1.5, *p* value < 0.05, q0 < 0.2 was considered as gain of copy number event in Nubian ibex, while CNV with normalized read depth < 0.7, *p* value < 0.05, and q0 < 0.2 was considered as loss of copy number event in Nubian ibex. CNV calls shared across the Nubian ibex individuals with > 50% overlap were retained. Finally, the DNA sequences spanning the candidate reference gain of copy number events were extracted from the domestic goat reference genome and subjected to dot plot analysis (using online dot plotter at NCBI; https://www.ncbi.nlm.nih.gov/) to test for tandem repeats or otherwise aligned to the entire genome of the domestic goat to detect segmental duplication using blast (parameters set to *e* value; 1e-10, percentage identity > 80%) (Altschul et al. 1990).

To account for variable regions that are not exclusive to Nubian ibex, we downloaded the genome coordinates of CNVs that have been identified in goat populations (Di Gerlando et al. 2020; Guan et al. 2020) and extracted overlapping sites using bedtools intersect (Quinlan and Hall 2010). Copy number variable regions overlapping (> 10%) with domestic goat CNV sites were excluded from further analysis.

### Assessment of Read Depth Using Simulated CNV Events

CNVnator has been mostly applied to detect CNVs within species, and the interpretation framework is not clear when applied to CNV detection between species, albeit closely related ones. For example, in the default framework, a read depth of 0.5 or less is taken to indicate a loss of copy in the test genome; however, a read depth of less than 0.5 can also be due to other genotypes such as a duplication in the reference. We carried out a simulation experiment to determine suitable cut-offs for CNV events and test the sensitivity of the CNVnator in detecting gain of copy events in the reference genome. Briefly, we simulated two duplications sites per chromosome in the domestic goat reference genome located at coordinates where the genome coverage was equal to the average read depth (copy number neutral). One of the sites was simulated to reflect two tandem copies and the other site four copies of fragments ranging in size from 3000 to 176,000 bp. CNVnator was then run as described previously using the simulated domestic goat genome as the reference.

### Gene Content and Functional Annotation

Genomic positions of the breakpoints (start and end positions of a CNV) shared across three Nubian ibex individuals as detected by CNVnator were annotated using Variant Effect Predictor (VEP v.95) (McLaren et al. 2016) relative to the domestic goat reference genome. Each position was assigned Sequence Ontology terms, defining different genomic regions: coding, upstream, downstream, non-coding, intergenic, intronic, and untranslated (UTR) sequences regions. CNVs that overlapped with only the introns of a gene (does not overlap with any exon) were classified as intronic CNVs, while those overlapping with some exons were considered exonic CNVs. CNVs that covered entire genes were classified as whole gene CNVs.

Gene ontology (GO) terms assignments for the CNV genes were found by searching the genes in Ensembl Goat Genes v.97 using Biomart (Kinsella et al. 2011), and additional gene functions were sourced from the literature. Gene enrichment analysis was carried out using The Database for Annotation, Visualization, and Integrated Discovery (DAVID) version 6.8 (Dennis et al. 2003). Since very few genes in the domestic goat reference genome have been assigned gene ontology terms, goat Ensembl gene IDs were converted to the orthologous human Ensembl gene ID using Biomart (https://www.ensembl.org/biomart/). The corresponding human Ensembl gene IDs were used for gene enrichment analysis as described above.

## Results

### Copy Number Variants Identification

A total of 446 million, 549 million, and 781 million paired-end sequence reads obtained from Nubian ibex individuals sampled from Egypt, Saudi Arabia, and South Africa, respectively, were downloaded from the public database. Approximately 88% and 97% of the sequence reads for the Saudi Arabia and Egyptian Nubian ibex individuals were mapped to the domestic goat reference genome. Successfully mapped sequence reads yielded 27-fold and 26-fold coverage for Saudi Arabia and Egyptian Nubian ibex. More than 98% of the sequence reads of Nubian ibex obtained from South Africa mapped successfully onto specific sites of the reference genome, ARS1, of the domesticated goat, *C. hircus*, with coverage of 36x. A summary of Nubian ibex sequence data is provided in Online Resource 1.

### Simulated CNV Sites Detection

We simulated tandem repeat CNV at 58 sites, two each per autosome, 38–43 were identified by CNVnator across the three Nubian ibex genomes, implying a sensitivity of just over 66–75%. The read depths for the simulated CNVs sites were 0.5 or less, as expected. Crucially, the read depth for the simulated CNV with two tandem copies was approximately 0.5, while for those with four tandem copies it was 0.25. We observed that all simulated CNVs detected had a mean q0 value ranging from 0.7 to 0.99, indicating that any novel CNV site with a q0 score greater than 0.7 is a valid CNV in which the number of copies in the reference is more than one. The simulation showed that CNVnator has a poor accuracy of detecting CNV sites' boundaries, with calls being on average 1654 bp away from the actual positions.

### CNVs in Nubian ibex and Domestic Goat Genomes

**Fig. 1:**
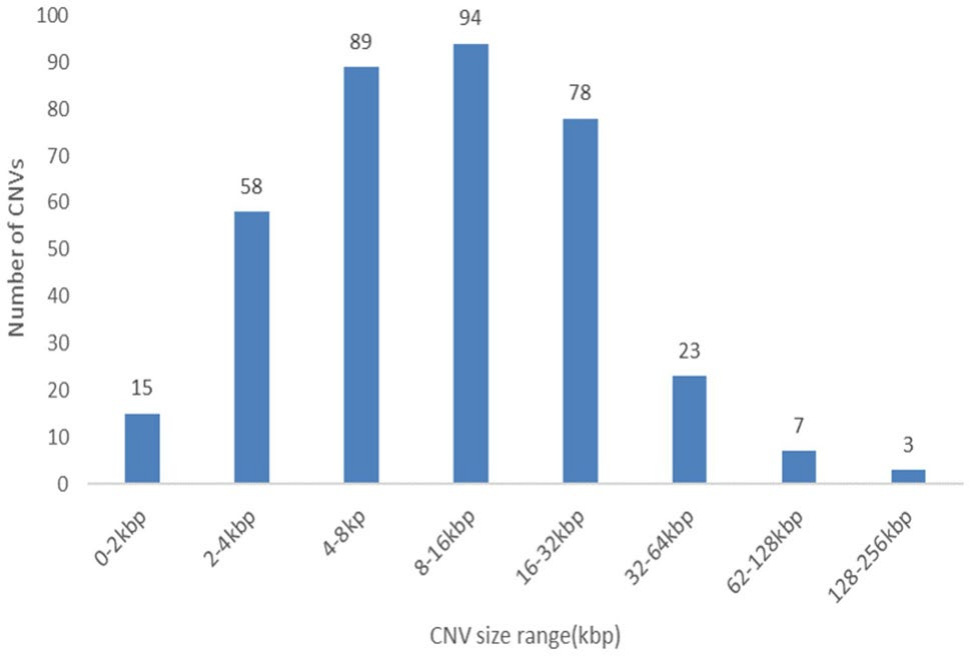
CNV size distribution across the Nubian ibex genome

CNVnator detected 13,472, 7724, and 9064 raw CNV loci in the Nubian ibex individuals obtained from South Africa, Egypt, and Saudi Arabia (see Online Resource 2). A total of 1726, 1544, and 1234 CNV loci detected in Nubian ibex individuals from South Africa, Egypt, and Saudi Arabia were retained after stringent filtering (see Online Resource 2). Twenty-seven CNVs detected previously in domestic goat genomes were further filtered out; refer to the Table in the Online Resources 3. Altogether 367 putative CNV loci were shared across the three individuals; 271 were gain of copy events in Nubian ibex and the domestic goat, while 96 were loss of copy events in Nubian ibex (see Table 1). The final CNV set of 367 loci covered less than 1% (5.6Mbp) of the Nubian ibex genome. The lengths of the CNVs ranged from 1.1 kbp to 214 kbp, with a median of 9.1 kbp (see Fig. 1). The estimated number of copies ranged between 0 and 463 copies.

**Table 1:** CNV loci detected in Nubian ibex individuals

CNV type	Nubian ibex sample source			CNV shared across Nubian ibexes
	South Africa (SA)	Egypt (E)	Saudi Arabia (A)	SA_E_A (intersect)
Deletions in Nubian ibex	971	750	682	97
Duplications in Nubian ibex	483	543	424	206
Duplication in reference	251	234	125	62
Duplications in reference genome with more copies in Nubian ibex genome	21	17	3	2
Total CNVs	1726	1544	1234	367

Deletions in Nubian ibex (Read depth < 0.7 *p* value < 0.05, q0 < 0.2); test genomes have few copy numbers relative to the reference genome. Duplication in Nubian ibex (Read depth > 1.5, *p* value < 0.05, q0 < 0.2); more copies in test genomes relative to the reference. Duplication in reference (Read depth < 0.7, *p* value < 0.05, q0 > 0.7); more copies in reference genome relative to the test genomes. Duplication in reference with more copies in Nubian ibex (RD > 1.20, *p* value < 0.05, q0 > 0.7); all genomes have many copies; however, the reference has more copy numbers than the test genomes

### CNVs Sequence Annotation

**Fig. 2:**
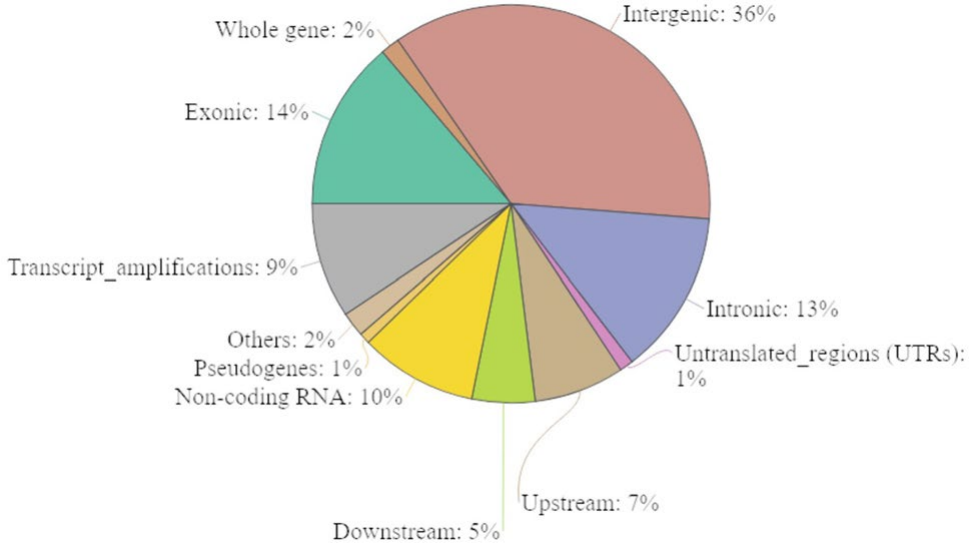
The distribution of 367 CNVs across the Nubian ibex genome (Intergenic, introns, exons, upstream/downstream, and untranslated regions). The majority of the CNVs were found in the intergenic regions of the genome

Sequence annotation showed that 161 (36%) of the CNVs were in intergenic regions. Other CNVs were in the genic regions, with 62 (14%) being exonic and 60 (13%) were intronic CNVs (see Fig. 2). CNV sequence annotation data is provided in Online Resource 4.

### Gene Content of the CNV Loci

The CNV events overlapped with 191 protein-coding genes, 9 lincRNAs, 7 snRNAs, 3 rRNAs, 4 pseudogenes, and 1 snoRNA. The CNVs spanning genes were outside the coding sequence regions (introns, upstream, downstream and untranslated gene regions) or within the coding sequence region (exons or entire genes). A total of 63 and 33 protein-coding genes overlapped with exonic and upstream region CNVs. Five whole genes overlapped with gain of copy number variable regions in Nubian ibex genome. Additionally, 60 intronic CNVs were reported, and 23 downstream CNVs (refer to Online resource 5). A summary of the number of protein-coding CNVs is provided in Table 2.

**Table 2:** CNV loci overlapping with protein-coding genes detected in Nubian ibex and domestic goat genomes

CNV type	CNV genes in coding sequence regions		CNV genes outside coding sequence region			
	Exonic	Whole gene	Introns	Upstream	Downstream	UTRs
Deletions in Nubian ibex	3	2	30	8	4	0
Duplications in Nubian ibex	54	5	22	22	19	4
Duplication in reference	6	0	8	3	0	1
Total CNV genes	63	7	60	33	23	5

Deletions in Nubian ibex; test genomes have few copy numbers relative to the reference genome. Duplication in Nubian ibex; more copies in test genomes relative to the reference. Duplication in reference; more copies in reference genome relative to the test genomes

**Fig. 3:**
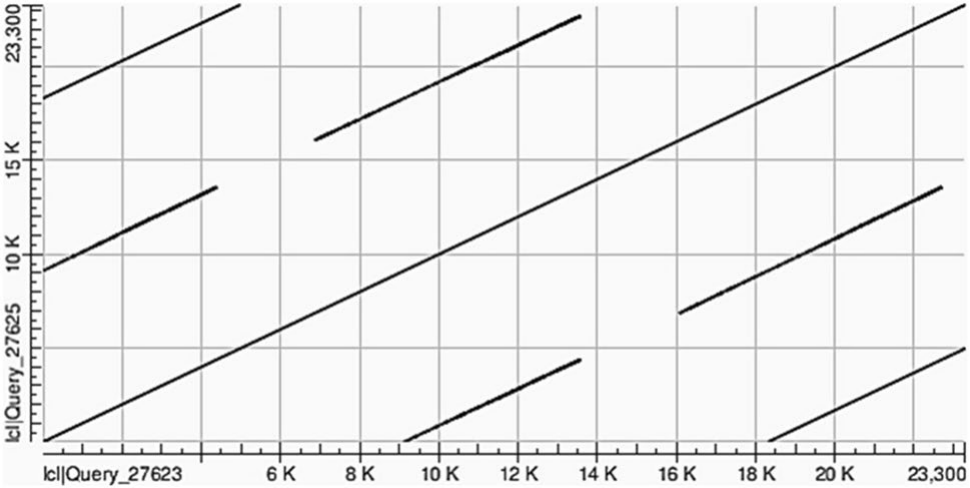
A dot plot of tandem duplication at chr1:67,890,500–67,913,800 in the domestic goat reference genome detected by CNVnator. The DNA sequence corresponding to the copy number variable region was extracted from the domestic goat reference genome using bedtools *–getfasta* tools (Quinlan and Hall 2010); the dot plot was generated from the sequence from alignment against itself in NCBI BLAST website (https://blast.ncbi.nlm.nih.gov/Blast.cgi). The CNV region overlaps with the first and the second exons of the Myosin Light Chain Kinase (*MYLK*) gene. The read depth of the CNV loci is 0.21, indicating that the domestic goat has four copies of the first two exons of *MYLK* gene, while the Nubian ibex has one copy

Of the protein-coding genes, 126 were in the gain of copy number variable regions, while 47 were in loss of copy number regions in Nubian ibex. Illustrations of gain and loss of copy events spanning protein-coding genes are provided in Online Resource 6. Eighteen protein-coding genes detected were in the gain of copy number variable regions in the domestic goat reference genome. Exonic gain of copy events in the domestic goat reference genome overlapped with six protein-coding genes, while intronic CNV overlapped with eight genes. Additionally, three protein-coding genes overlapped with upstream CNVs in the domestic goat reference genome. A dot plot of CNV event in the domestic goat reference genome with read depth < 0.7 and (q0 > 0.7) depicting tandem duplication is illustrated in Fig. 3.

### Functions of the CNV-Associated Protein-Coding Genes

Gene ontology assignments showed that the CNV-associated protein-coding genes are involved in diverse biological processes such as complement activation, inflammatory response, proteolysis, negative regulation of endopeptidase activity, drug metabolism, and positive regulation apoptotic cell clearance, among several other roles. No gene ontology (GO) terms were significantly enriched (See Online Resource 5).

Gene functional analysis showed that the CNV-associated protein-coding genes are involved in diverse biological processes including innate immune response (Cluster of Differentiation 54 (CD54)), Interferon Beta 1(IFNB1)), Intercellular adhesion molecule-1 (ICAM-1), bovine major histocompatibility complex (BoLA), Interferon-induced transmembrane protein 3 (IFITM3) and Complement Factor H Related 4 (CFHR4)). Other copy number variable immune genes reported include: UL16 binding protein 3 (ULBP3), Natural Killer Group 2D ligand 4-like (NKG2D ligand 4-like), NKG2D ligand 1-like (NKG2D ligand 1-like), Bactericidal/permeability-increasing fold containing family A, member 1 (BPIFA1), Cluster of Differentiation 48 (CD48), complement C3 (C3), complement C4B (Chido blood group) (C4B), Cluster of Differentiation 54 (CD54), adhesion G protein-coupled receptor E3 (ADGRE3) and serpin family A member 3 (SERPINA3) (See Table 3).

**Table 3:** Protein-coding genes overlapping with CNVs within the exons and upstream gene regions

Biological process	Gain of copy number genes in Nubian ibex (RD > 1.5, *p* < 0.05, q0 < 0.2)	Gain of copy number genes in the domestic goat (RD < 0.7, *p* < 0.05 > 0.7)
Detoxification/xenobiotic metabolism	GSTM4, CYP2D14, CYP2B6, CYP2D6, CYP2C31, UGT2B31, MRP4Like, MRP4, PHYH, CES1, UGT2B7	–
Innate immunity	GSTM4, GIMAP7, CD48, C3, NKG2D ligand 4-like, NKG2D ligand 1-like,ULBP3, BPIFA1, MYADM, SERPIN A3-6, SERPIN B6, C4A, C4B, IFNB1, CD54, CFHR4, UBD, SIGLEC5, KIR2DL1, TNFRSF10B-like, CD163L1, ADGRE3, TRAV22, IGLV5-45, IGLV1-40, IFITM3, BoLA, Antigen WC1.1-like, IFI27L2	ADGRE2
Cell growth, recycling and metabolism	GIMAP7, LRP11L, PHYH, ZYMND8, MYADM, NPC1, RMC1, POLB, PIK3R4, C19orf12, UBD	TBC1D22B
Lipid and energy metabolism	LRP11L, MRP4, MRP4L, PCSK2, FADS2L, ACOT13, CFHR4, C19orf12, NPC1, LRP11, MT-ATP6	–
Fertility/reproduction	MAN2B2, MAN2B2-Like, CSH2, MYADM, ADAM18	DDX25, CELSR1
Other	CYP21A2, KRTAP1-1, GPR157, STK19, PINLYP, C19orf12, OR2H1D, C6orf62, PRDM9, SLC2A5	KRTAP3-1, MYLK, KRTAP1-1, PRKCA, CRYM-AS1, TBC1D22B

Similarly, xenobiotic and drug metabolic process genes (carboxylesterase 1 (CES1), cytochrome P450 family 2 subfamily B member 6 (CYP2B6), Cytochrome P450 2D6 (CYP2D6), cytochrome P450 family 2 subfamily D member 14 (CYP2D14), Glutathione S-transferase Mu 4 (GSTM4), UDP-glucuronosyltransferase 2B31 (UGT2B31) UDP-Glucuronosyltransferase-2B7 (UGT2B7), phytanoyl-CoA hydroxylase (PHYH), and Multidrug resistance protein 4 (MRP4) were reported to be copy number variable in Nubian ibex. Furthermore, lipid and energy metabolism genes such as low-density lipoprotein receptor-related protein 11(LRP11-like), Acyl-CoA thioesterase 13 (ACOT13), Mitochondrial Encoded ATP Synthase Membrane Subunit 6 (MT-ATP6), and Fatty acid desaturase 2 (FADS2) were shown to be copy number variable in Nubian ibex genome. Other CNV-associated genes reported are involved in reproductive functions such as MAN2B2, MAN2B2-Like, CSH2, MYADM, ADAM18 in Nubian ibex and DDX25 in domestic goat. A summary of genes overlapping with CNVs in the exons and upstream gene regions is provided in Table 3 and Online Resource 7.

## Discussion

### Copy Number Variants Calling

CNVs were inferred from sequence data of three Nubian ibexes relative to the domestic goat reference genome using read-depth approach (Abyzov et al. 2011). Read depth is a well-established approach whose sensitivity and power to detect CNVs have been confirmed experimentally (Paudel et al. 2015; Pezer et al. 2015; Wang et al. 2019). For example, assessment of read-depth using droplet digital PCR (ddPCR) showed that the CNVs detected using ddPRC strongly correlated with those identified using a read-depth approach with a low false discovery rate of 8.7% (Pezer et al. 2015). Based on previous experimental validation of the read-depth method, we did not confirm the CNVs identified in this study; hence, the results should be considered in light of this limitation. However, our simulation experiment carried out by introducing ‘artificial CNVs’ to known regions in the reference genome showed that CNVnator could detect approximately 71% of the simulated CNVs, thus supporting that most of the CNVs detected in our study are potential true positives. Our simulation experiment showed that CNVnator has poor accuracy in detecting CNV site boundaries; this limitation could lead to overestimation or underestimation of CNVs. In total, 1234, 1544, and 1726 CNV loci were detected in the three analyzed Nubian ibexes, a number which is comparable to CNVs discovered in other livestock species such as yak (Zhang et al. 2016). Altogether, 367 CNVs were detected across the Nubian ibex genomes. Twenty-seven (27) CNVs identified across the Nubian ibexes reported previously in domestic goat genomes (Di Gerlando et al. 2020; Guan et al. 2020) were discarded since they reflect polymorphisms in/across goat species. Although only three Nubian ibexes were investigated, the samples were derived from unrelated individuals representing different geographical populations (South Africa, Egypt and Saudi Arabia). Therefore, the identified overlapping CNVs potentially represent Nubian ibex specific CNVs.

### Copy Number Variable Protein-Coding Genes

Several protein-coding genes overlapping with copy number variable regions were reported in the three Nubian ibexes and domestic goat genome, representing a valuable resource for future studies on the relation between CNV genes and phenotypic variations. Twenty GO terms were associated with the protein-coding CNV genes; however, none were significant in terms of enrichment. Nevertheless, the gene ontology assignment indicated that the CNV-associated protein-coding genes are involved in diverse biological processes such as cell growth, recycling and metabolism, and energy metabolism. Consistent with other CNV studies in livestock species (Bickhart et al. 2012; Zhang et al. 2016), we found clusters of drug metabolism and immune-related genes in copy number variable regions in the Nubian ibex genome.

### The Possible Roles of CNV Genes in Nubian ibex Adaptations

We found clusters of CNV-associated protein-coding genes involved in immune response and drug metabolisms that deserved more attention because of their known functions. Genes involved in defense response to bacterial or viral infections such as BPIFA2, CD48, ULBP3, NKG2D ligand 1-like, and NKG2D ligand 4-like were reported to have more copies in the genomes of Nubian ibex relative to the domestic goat reference. BPIFA2 is a glycoprotein expressed in the airway epithelium and submucosal glands of the upper airways, where it offers protection against bacterial and viral infections (Akram et al. 2018; Liu et al. 2013). Previous studies have shown that BPIFA2 has a defensive role in *K. pneumoniae* infection virus (Zhou et al. 2008), suggesting that BPIFA2 might have defensive roles against other viruses. Gain of copy number variations of CD48, a member of the signaling lymphocyte activation molecular family, was observed in the investigated Nubian ibexes. CD48 is involved in diverse immune responses ranging from T cell activation, granulocyte activity, allergic inflammation to natural killer function and antimicrobial immunity (McArdel et al. 2016). Although CD48 plays diverse immune response roles, studies have shown that it is a target of immune evasion by viruses (McArdel et al. 2016). The mucin-like protein m153 in murine cytomegalovirus (CMV) has been shown to reduce expression of CD48 on macrophages, limiting NK-cell-mediated control of viral infection (Zarama et al. 2014). We hypothesize that the gain of copy number variation in CD48 might be an evolutionary adaptation that enables it to produce diverse functional proteins to confer the needed immunosurveillance. Our results showed that NKG2D ligands system genes (ULBP3, NKG2D ligand 1-like, and NKG2D ligand 4-like) were under gain of copy events in Nubian ibex. ULBP3 and NKG2D ligand 4-like expressions are induced by stressors, such as viral infections, heat shock, tissue damage, tumorigenesis, and DNA damage (Lanier 2015). Once expressed, NKG2D ligands bind to NKG2D receptors, which mounts cell-mediated cytotoxicity and cytokine production, thus eliminating stressed cells (Zingoni et al. 2018). Nubian ibex is exposed to viral diseases such as the Peste des petits ruminants virus (Clarke et al. 2017; Wensman et al. 2018) and Malignant catarrhal fever virus (Okeson et al. 2007) in its environment. The exonic gain of copy numbers immune genes (BPIFA1, CD48, ULBP3, NKG2D ligand 1-like, and NKG2D ligand 4-like) could be an evolutionary mechanism that enables Nubian ibex to encode functionally diverse proteins through alternate splicing in response to viral stressors.

Other immune response genes reported include C3, C4A, and C4B that play essential roles in the activation of the classical and lectin pathways of the complement system that lead to neutralization of invading microbes and clearance of immune complexes (Miyagawa et al. 2008; Yang et al. 2007). Deficiency of the C3 gene is associated with susceptibility to systemic lupus erythematosus (SLE) (Miyagawa et al. 2008). Similarly, many copy numbers of C4 have been shown to alleviate susceptibility to systemic lupus erythematosus (SLE) in humans (Jüptner et al. 2018). Other recent studies have shown that increased copy numbers of C4A offer protection against age-related macular degeneration (AMD) (Grassmann et al. 2016). Further studies are needed to ascertain the possible role of complement component genes (C3, C4A, and C4B) in Nubian ibex adaptations; however, it is known to play critical roles in immunosurveillance. Clusters of immune response genes may reflect the different immune response strategies between the Nubian ibex and the domestic goat in response to pathogens in their environments.

Xenobiotic metabolism genes including CYP2D14, CYP2B6, CYP2D6, CYP2C31, UGT2B31, UGT2B7, GSTM4, CES1, MPRP4, and MRP4Like were shown to be under the gain of copy number variations in Nubian ibex. Drug-metabolizing genes play crucial roles in eliminating plant secondary metabolites that could otherwise be toxic to livestock species (Maréchal et al. 2008). The cytochromes P450 genes (CYP2D14, CYP2B6, CYP2D6, and CYP2C31) and Carboxylesterase (CES1) are oxidizing enzymes that mediate biotransformation of xenobiotic compounds(phase I reactions) (Maréchal et al. 2008; Wang et al. 2018). The UDP-glucuronosyltransferases (UGT2B7 and UGT2B1) and glutathione S‐transferases (GSTM4), on the other hand, are conjugative enzymes that play a vital role in glucuronidation (phase II) (Iyanagi 2007). The conjugated compounds with glucuronate or glutathione are transported out of the cell by the ATP-binding cassette transporters (ABC transporters) such as MRP4Like and MRP4 (Russel et al. 2008). Desert plants are rich in secondary metabolites such as alkaloids, flavonoids, oxalates, and tannins (Jacobson et al. 2009; Robertson et al. 2018; Zahedi et al. 2019). Nubian ibex have evolved in deserts, and it has been observed that they consume alkaloid-producing plants when food is scarce (Habibi 1994; Hakham and Ritte 1993). Gain of copy numbers of xenobiotic metabolisms genes could be one of the evolutionary mechanisms which enable the Nubian ibex to cope with the toxic secondary metabolites in their diet.

## Conclusion

In this study, we compared the genome of the endangered desert-adapted Caprine species (Nubian ibex) with that of the domesticated goat (*C. hircus*), which is of substantial economic importance globally. We sought to gain insights from the copy number variants at the genome-wide level and their possible role in adaptations of the Nubian ibex to the desert habitat. A total of 367 CNV loci shared across three analyzed Nubian ibex individuals were identified as potential CNV sites, many of which overlapped known protein-coding genes. From the analysis of the involved genes, we conclude that potentially a significant driver for the difference between domestic goat and the Nubian ibex is the response to viral disease burdens and plant secondary metabolites in their diet as indicated by a preponderance of genes involved in response to viral infections and xenobiotic metabolisms among the CNV loci. This study is exploratory and provides a basis for further evolutionary studies in Nubian ibex genome.

## Supplementary Information

Below is the link to the electronic supplementary material.

Supplementary file1 (CSV 121 kb) Raw and filtered CNVs detected across the three analyzed Nubian ibex genomes

Supplementary file2 (CSV 98 kb) Raw and filtered CNVs detected across the three analyzed Nubian ibex genomes

Supplementary file3 (CSV 123 kb) Raw and filtered CNVs detected across the three analyzed Nubian ibex genomes

Supplementary file4 (DOCX 12 kb) Nubian ibex sequence data summary. The file is a summary of the sequence data used for CNV calling

Supplementary file5 (XLSX 10 kb) CNVs detected in Nubian ibexes’ genomes that overlap with CNVs detected in domestic goats’ genomes.

Supplementary file6 (XLSX 62 kb) The file contains CNVs sequence annotations

Supplementary file7 (XLSX 53 kb) CNVs associated protein-coding genes and their corresponding gene ontology analysis

Supplementary file8 (PDF 604 kb) Illustrations of gain and loss of copy number events in Nubian ibex genome

Supplementary file9 (DOCX 20 kb) CNVs found upstream and in exonic regions of protein-coding genes

Supplementary file10 (CSV 515 kb) Raw and filtered CNVs detected across the three analyzed Nubian ibex genomes

Supplementary file11 (CSV 577 kb) Raw and filtered CNVs detected across the three analyzed Nubian ibex genomes

Supplementary file12 (CSV 835 kb) Raw and filtered CNVs detected across the three analyzed Nubian ibex genomes

## Data Availability

The sequence data (FASTQ) files used in this study were obtained from public databases under Accession (SRR12990712, SRR8437789 and SRR8437792). Other CNV data generated in this study are provided at Figshare (https://figshare.com/articles/dataset/Copy_number_variants_in_Nubian_ibex/13633943).
