# Supplementary material for: Genome Scan for Variable Genes Involved in Environmental Adaptations of Nubian Ibex
Source: J Mol Evol. 2021 Jun 17;89(7):448–57. doi: 10.1007/s00239-021-10015-3 (PMC8318948; doi:10.1007/s00239-021-10015-3)
Supplement: Supplementary file 4 — Supplementary file4 (DOCX 12 kb) Nubian ibex sequence data summary. The file is a summary of the sequence data used for CNV calling [file 239_2021_10015_MOESM4_ESM.docx]

**Table 1: Nubian ibex sequence data statistics**

| Nubian ibex sample source | Downloaded sequence reads | Sequence reads after quality control | Coverage for filtered reads | Mapping to domestic goat |
| --- | --- | --- | --- | --- |
| South Africa | 781,955,700 | 781,955,700 | 36x | 769,628,812 (98%) |
| Egypt | 446,250,278 | 446,250,278 | 20x | 432,862,770 (97%) |
| Saudi Arabia | 556,887,563 | 549,192,312 | 19x | 483,289,235 (88%) |
