# Supplementary material for: Genome Scan for Variable Genes Involved in Environmental Adaptations of Nubian Ibex
Source: J Mol Evol. 2021 Jun 17;89(7):448–57. doi: 10.1007/s00239-021-10015-3 (PMC8318948; doi:10.1007/s00239-021-10015-3)
Supplement: Supplementary file 8 — Supplementary file8 (PDF 604 kb) Illustrations of gain and loss of copy number events in Nubian ibex genome [file 239_2021_10015_MOESM8_ESM.pdf]

Title: Genome Scan for Variable Genes Involved in Environmental Adaptations of Nubian Ibex

Authors: Vivien J. Chebii, Emmanuel Mpolya, Samuel O. Oyola, Antoinette Kotze , Jean-Baka Domelevo Entfellner, J. Musembi Mutuku

Journal: Journal of Molecular evolution

Corresponding author information

Vivien J Chebii

Corresponding author: Email: chebiiv@nm-aist.ac.tz; chevivien@gmail.com

School of Life Science and Bioengineering, Nelson Mandela African Institution of Science and Technology, P.O. Box 447, Arusha, Tanzania.

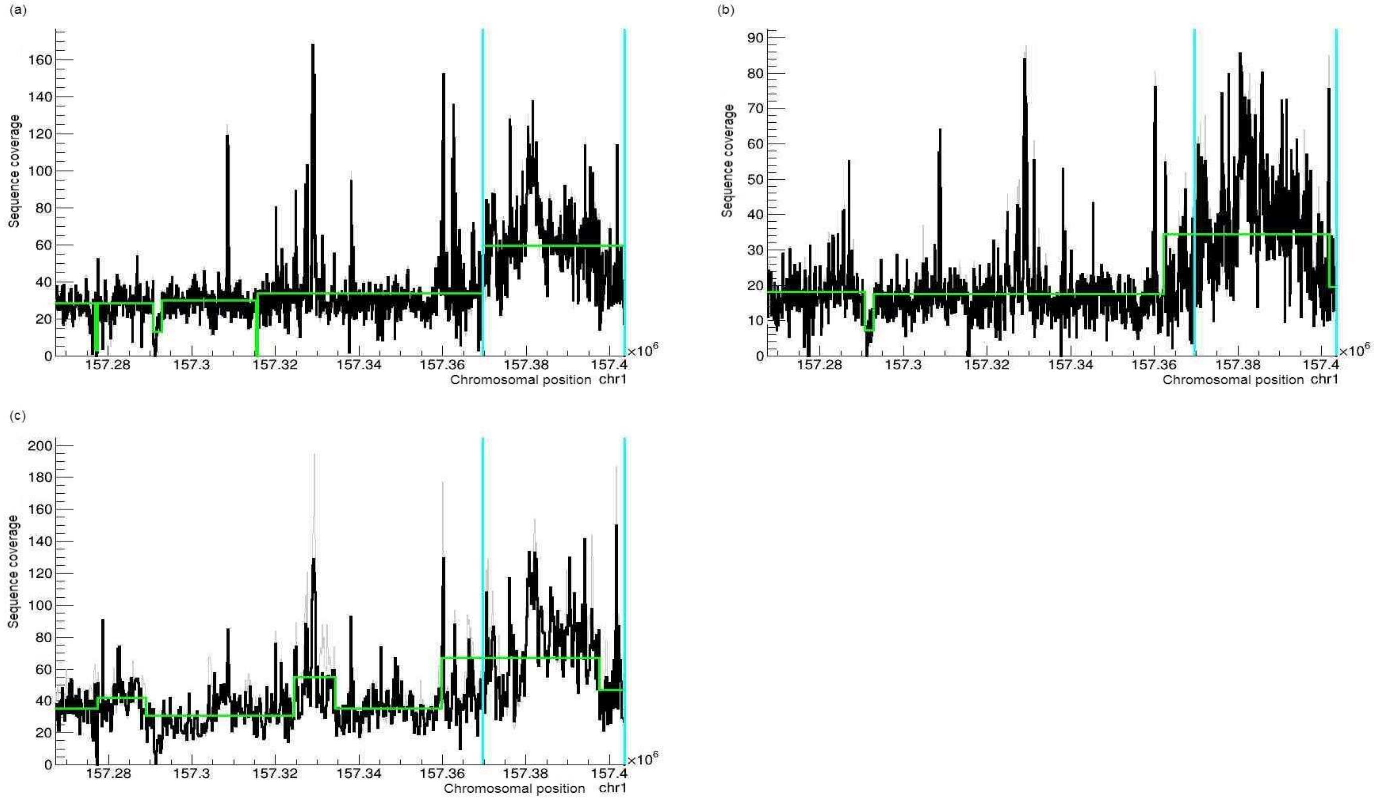

Fig. 1. Read depth plots against chromosome 1 illustrating gain of copy number event. The read depth plots were generated using CNVnator –view program (Abyzov et al., 2011); (a) is read depth plot generated from sequence data for Nubian ibex sampled South Africa, (b) Egypt and (c)Saudi Arabia. The green lines indicate normalized read depth, while the section enclosed in blue vertical lines depicts gain of copy number region (chr1:157369600-157403600) shared across the three analyzed Nubian ibexes. The gain of copy number region overlaps PRDM9 gene, which is found at chr1:157367662-157383070:1.

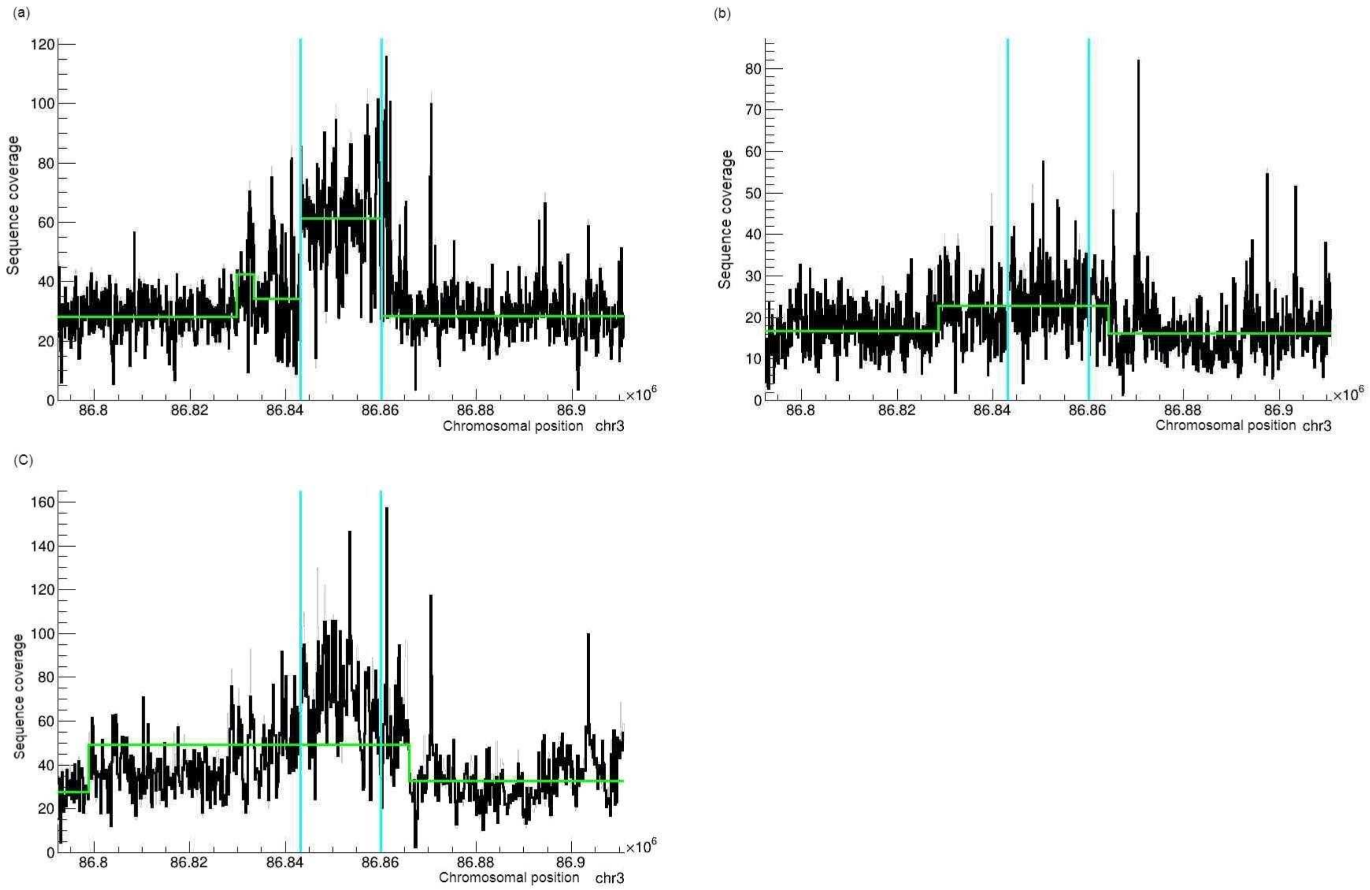

Fig. 2. Read depth plots against chromosome 3 illustrating gain of copy number event. The read depth plots were generated using CNVnator –view program (Abyzov et al., 2011); (a) is read depth plot generated from sequence data for Nubian ibex sampled from South Africa, (b) Egypt and (c)Saudi Arabia. The green lines indicate normalized read depth, while the section enclosed in blue vertical lines depicts gain of copy number region (chr3:86843200-86860100) shared across the three analyzed Nubian ibexes. The gain of copy number region overlaps the first two exons of GSTM4 gene, which is found in chr3:86846458-86878863.

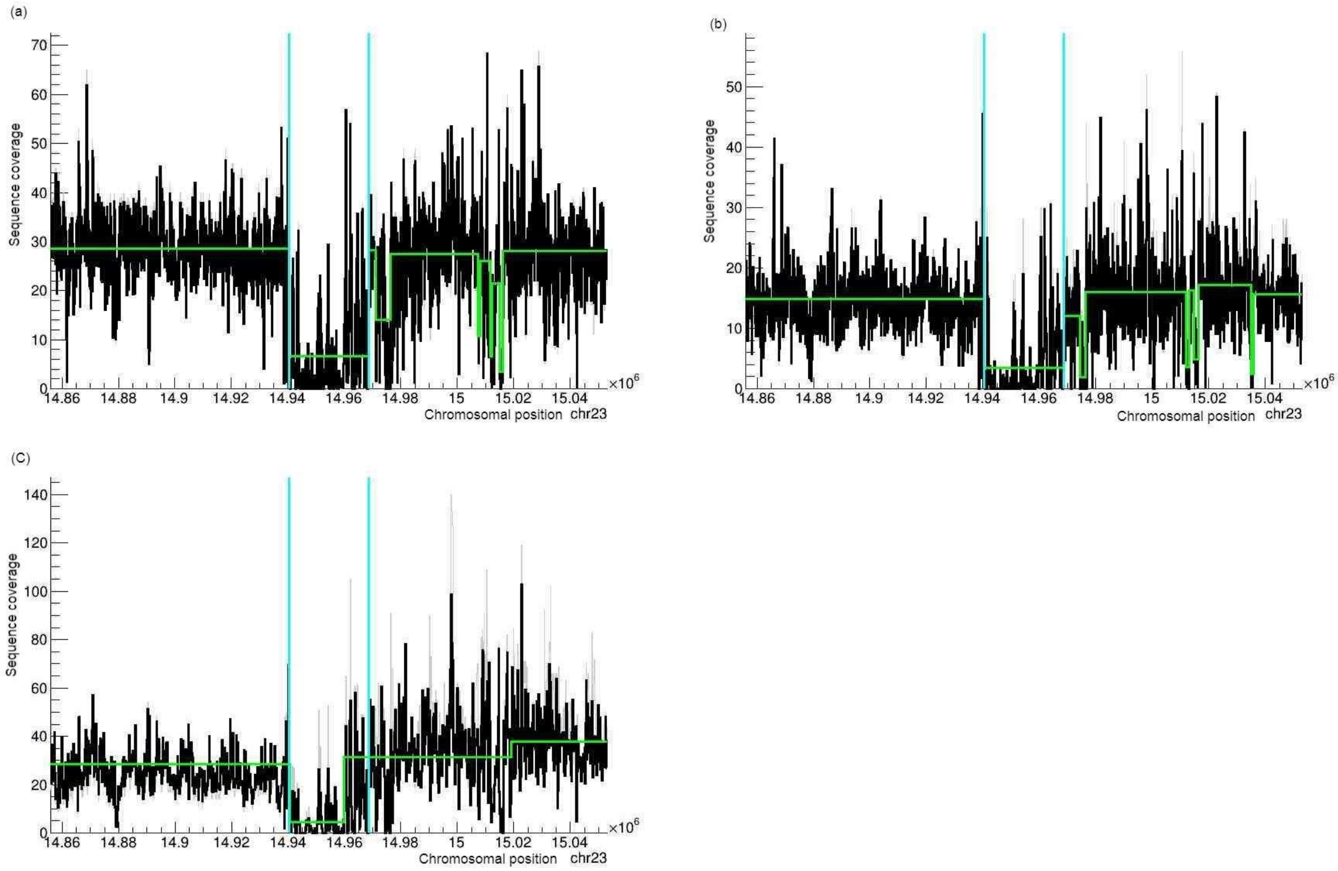

Fig. 3. Read depth plots against chromosome 23 illustrating the loss of copy number event. The read depth plots were generated using CNVnator –view program (Abyzov et al., 2011); (a) is read depth plot generated from sequence data for Nubian ibex sampled South Africa, (b) Egypt and (c)Saudi Arabia. The green lines indicate normalized read depth, while regions enclosed in blue vertical lines depict a loss of copy number region (chr23:14940500-14968600) shared across the three analyzed Nubian ibexes. The loss of copy number region overlaps with Serpin B6-like gene, which is found at chr23:14938589-14955225: -1

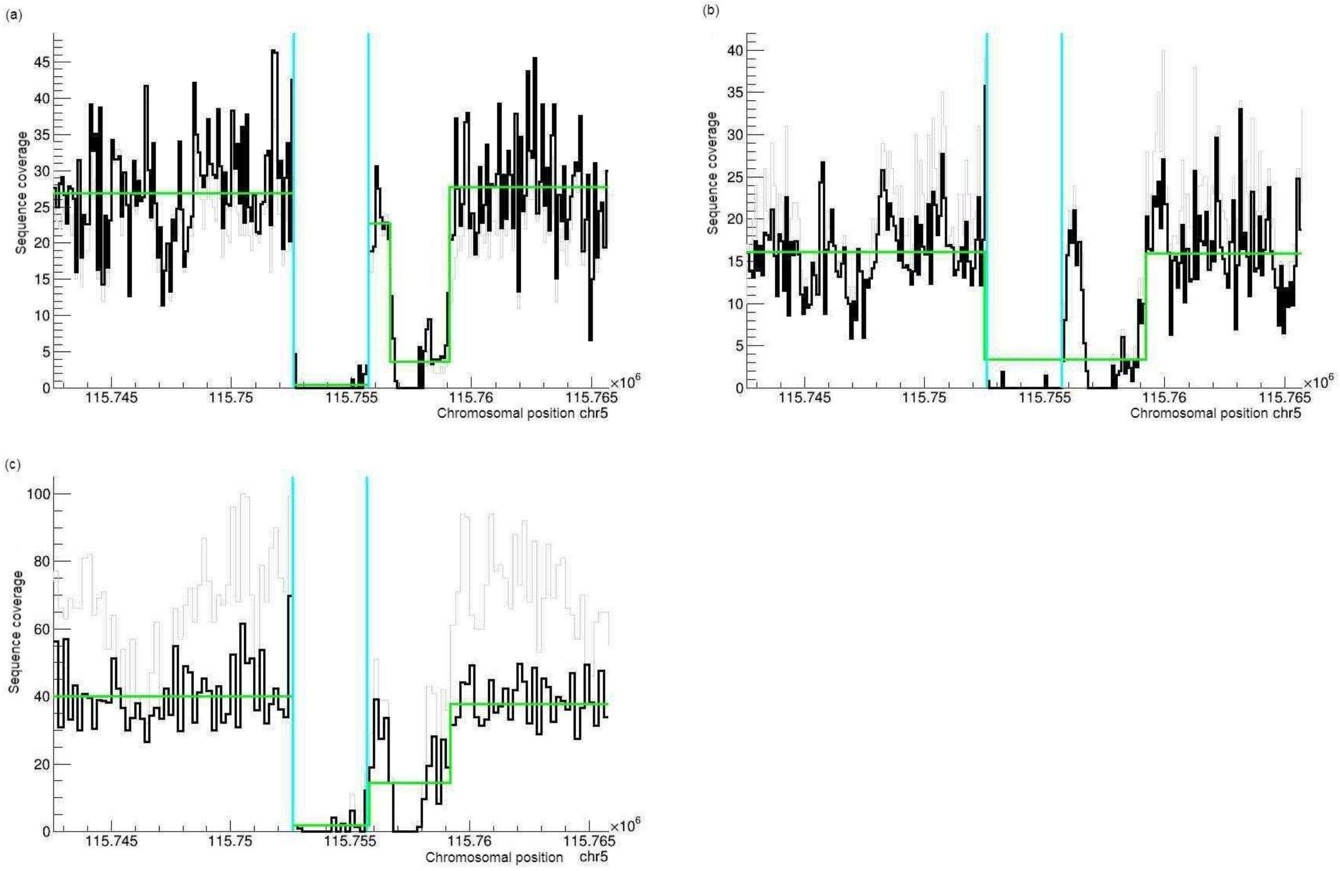

Fig. 4. Read depth plots against chromosome 5 illustrating a loss of copy number event. The read depth plots were generated using CNVnator –view program (Abyzov et al., 2011); (a) is read depth plot generated from sequence data for Nubian ibex sampled from South Africa, (b) Egypt and (c)Saudi Arabia. The green lines indicate normalized read depth, while the section enclosed in blue vertical lines depict a loss of copy number region (chr5:115752600-115755700) shared across the three analyzed Nubian ibexes. The loss of copy number region overlaps the first two exons of CELSR1 gene, which is found at chr5:115594667-115755126: -1.
