# Supplementary material for: Genome Scan for Variable Genes Involved in Environmental Adaptations of Nubian Ibex
Source: J Mol Evol. 2021 Jun 17;89(7):448–57. doi: 10.1007/s00239-021-10015-3 (PMC8318948; doi:10.1007/s00239-021-10015-3)
Supplement: Supplementary file 9 — Supplementary file9 (DOCX 20 kb) CNVs found upstream and in exonic regions of protein-coding genes [file 239_2021_10015_MOESM9_ESM.docx]

**Table 1A CNVs within upstream of protein-coding genes**

| Co-ordinates | Gene symbol | Read depth | Function |
| --- | --- | --- | --- |
| chr1:143938000-143943000 | PIK3R4 | 3.79449 | Protein phosphorylation |
| chr3:86843200-86860100 | GSTM4 | 2.16326 | Xenobiotic catabolic process |
| chr5:101608200-101629300 | Antigen WC-7 | 2.48411 | Immune response |
| chr7:94689600-94694400 | CD54 | 1.92372 | Immune response |
| chr7:96343900-96350900 | Pcp2 | 0.150812 | unknown |
| chr7:96343900-96350900 | XAB2 | 0.150812 | Transcription-coupled DNA repair |
| chr8:22709200-22715600 | WC-1 | 3.08422 | Immune response |
| chr8:23058100-23116600 | IFNB1 | 3.37647 | Immune response |
| chr8:23133700-23137800 | IFNA2 | 2.09312 | Immune response |
| chr9:74409600-74415400 | LRP11 | 2.07603 | Lipid metabolism |
| chr10:78472100-78477100 | TRAV22 | 1.72914 | Immune response |
| chr14:4908500-4915500 | CA1-like | 0.396292 | one-carbon metabolic process |
| chr15:32435400-32450300 | novel gene | 0.280498 | Not available |
| chr15:35993300-36013100 | GVINP1-like | 2.20337 | Immune response (pseudogene) |
| chr16:5137400-5142700 | CFHR4 | 1.80752 | Complement activation |
| chr16:49628400-49630300 | MRPL20 | 0.0035275 | ribosomal large subunit assembly |
| chr17:645700-647900 | IGLV5-45 | 1.94319 | Immune response |
| chr17:645700-647900 | IGLV5-45 | 1.94319 | Immune response |
| chr17:68517000-68531000 | GUCY1B1 | 463.908 | cGMP biosynthetic process |
| chr18:52827500-52831900 | PINLYP | 0.284695 | negative regulation of catalytic activity |
| chr18:59974600-59980700 | GNB1 | 1.61126 | signal transduction |
| chr18:59974600-59980700 | ZNF345 | 1.61126 | transcription by RNA polymerase II |
| chr18:60145500-60153700 | ZNF665-like | 1.62231 | transcription by RNA polymerase II |
| chr19:40856200-40871000 | KRTAP3-1 | 0.16045 | Hair shaft development |
| chr19:61931400-61955000 | PRKCA | 0.313423 | protein phosphorylation |
| chr23:20958900-20972000 | BoLA | 1.85886 | Immune response |
| chr23:20958900-20972000 | IFITM3 | 1.85886 | Immune response |
| chr23:20994300-21003800 | UBD | 1.56011 | Protein ubiquitination, immune response |
| chr23:20994300-21003800 | OR2H2 | 1.56011 | Signal transduction, olfactory transduction |
| chr23:21030300-21048500 | C19orf12 | 1.9336 | Apoptotic process, response to oxidative stress |
| chr23:22598000-22604400 | CYP21A2 | 1.87191 | Sterol metabolic process, oxidation-reduction process |
| chr23:37615100-37624900 | TBC1D22B | 0.184295 | Activation of GTPase activity |
| chr26:369700-371000 | ZNF511 | 0.0210928 | Unknown |
| chr29:46015400-46028600 | PKP3 | 0.0040550 | positive regulation of gene expression |

Positions of the CNV loci overlapping with upstream gene regions and the corresponding genes. Read depth>1.5=duplication in Nubian ibex, read depth<0.8=duplication in the reference genome.

**Table 1B CNVs spanning exonic regions of genes in Nubian ibex genome**

| **Co-ordinates** | **Read depth** | **Gene symbol** | **Exons affected** | **Function** |
| --- | --- | --- | --- | --- |
| chr1:67890500-67913800 | 0.211003 | MYLK | 1-2/31 | Regulates actin-myosin interaction of smooth muscle |
| chr3:20800-47300 | 4.42965 | Novel gene | 1-8/13 | Not available |
| chr3:21602000-21616700 | 1.91448 | CYP4A21 | 1-14/15 | Bile acid biosythesis |
| chr3:86843200-86860100 | 2.16326 | GSTM4 | 1-2/8 | Xenobiotic catabolic process |
| chr3:93562700-93573400 | 0.209682 | DDX25 | 1-4/5 | Regulation of translation,  Reproduction |
| chr3:111010400-111025800 | 2.09274 | CD48 | 2/4 | Immune response |
| chr4:7003000-7019400 | 1.80589 | GIMAP7 | 2/2 | GTP metabolic process |
| chr5:99356100-99360700 | 1.82596 | Antigen WC1.1-like | 3-4/19 | Immune response |
| chr5:101608200-101629300 | 2.48411 | CD163L1 | 5-18/18 | Immune response |
| chr5:112038800-112048300 | 1.55899 | CYP2D14 | 5-9/9 | Xenobiotic metabolic process |
| chr5:112058600-112068400 | 1.78983 | CYP2D6 | 2-10/10 | Xenobiotic metabolic process |
| chr5:115752600-115755700 | 0.0159134 | CELSR1 | 1-2/36 | Reproduction |
| chr6:5876900-5889700 | 2.45276 | ARHGAP20 | 20-24/26 | Blood pressure regulation |
| chr6:85040500-85060000 | 2.55871 | UGT2B31 | 1-3/6 | Xenobiotic glucuronidation |
| chr6:113107600-113143400 | 2.00817 | MAN2B2 | 1-14/22 | Mannose metabolic process |
| chr6:113244100-113248600 | 1.85053 | MAN2B2-like | 13-16/19 | Mannose metabolic process |
| chr7:61279200-61290500 | 252.225 | MT-ATP6 | 1/4 | Energy metabolism |
| chr7:92685000-92733800 | 1.89 | C3 | 3-41/41 | Immune response, complement activation |
| chr7:93096500-93109400 | 1.80499 | ADGRE3 | 11-16/17 | Immune response |
| chr7:96343900-96350900 | 0.150812 | ADGRE2 | 1-5/18 | Immune response |
| chr8:58914800-58989000 | 1.877899 | FAM205A | 2/2 | Not available |
| chr9:74011600-74040500 | 4.26357 | NKG2D ligand 1-like | 2-5/5 | Natural killer cell-mediated cytotoxicity |
| chr9:74047100- 74260900 | 3.16891 | NKG2D ligand 4-like | 1-3/4 | Natural killer cell-mediated cytotoxicity |
| chr9: 74316601 -74455600 | 2.0597 | ULBP3 | 2-5/5 | Natural killer cell-mediated cytotoxicity |
| chr9:74459600-74469600 | 2.19994 | LRP11-like | 1-5/6 | Multicellular organismal response to stress |
| chr12:14187699:14206800 | 2.836327 | MRP4-like | 1-3/31 | Xenobiotic organic anionic compounds transporter |
| chr12:14851899:14991100 | 2.004655 | MRP4 | 3-29/30 | Xenobiotic organic anionic compounds transporter |
| chr13:27293500-27299000 | 1.80671 | PHYH | 7-9/9 | Fatty acid alpha-oxidation |
| chr13:37185999:37258900 | 1.876872 | PCSK2 | 4-8/8 | Nervous system development, |
| chr13:37881500-37887500 | 1.91498 | DZANK1 | 16/17 | Eye photoreceptor cell development |
| chr13:62201600-62225800 | 2.00343 | BPIFA2 | 1-4/6 | Defense response to bacterial and viral infections |
| chr13:75273299:75330600 | 3.179351 | ZMYND8 | 11-21/21 | DNA repair |
| chr15:3170000-3175100 | 3.17816 | KRTAP1-1 | 1/11 | Hair shaft development |
| chr15:3960600-4043600 | 3.556838 | FADS2-like | 1-5/11 | Lipid metabolic process |
| chr16:924300-934300 | 1.62388 | ATP2B4-like | 2-6/6 | Regulation of sodium ion transmembrane transport |
| chr16:5911800-5935000 | 1.975405 | ATP2B4 | 2-15/22 | Regulation of sodium ion transmembrane transport |
| chr16:42908700-42931800 | 1.94538 | GPR157 | 3-4/4 | neuronal differentiation of radial glial progenitors |
| chr16:42933800-42946600 | 1.79468 | SLC2A5 | 1/12 | Fructose transporter |
| chr18:1793200-1808200 | 1.89604 | CSH2 | 1/5 | Growth control |
| chr18:26110200-26128700 | 2.29226 | CES1 | 2-9/13 | Xenobiotic metabolic process |
| chr18:51520800-51541500 | 1.86789 | CYP2B6 | 1/9 | Xenobiotic metabolic process |
| chr18:60212000-60233600 | 2.21944 | ZNF501 | 2/2 | Regulation of transcription |
| chr19:40893400-40912100 | 0.439653 | KRTAP1-1 | 3/3 | Hair shaft development |
| chr19:61931400-61955000 | 0.313423 | PRKCA | 1/15 | Positive regulation of endothelial cell proliferation |
| chr21:19029800-19050200 | 3.35655 | MYADM | 2-3/4 | Positive regulation of cell migration |
| chr21:58140600-58166700 | 1.99648 | IFI27L2 | 2-5/5 | Immune response |
| chr21:58721700-58729200 | 1.86488 | SERPINA3-6 | 3-4/4 | Inflammatory response |
| chr23:14940500-14968600 | 0.23343 | Serpin B6 | 4-7/7 | cellular response to osmotic stress |
| chr23:16889100-16898100 | 2.00543 | ACOT13 | 1-2/3 | Long-chain fatty-acyl-coa biosynthetic process |
| chr23:22585900-22604400 | 2.04911 | STK19  C4A  C4B | 6-7/7  1-17/44  45/45 | Protein phosphorylation  Innate immune response, complement activation, inflammatory response, |
| chr24:33378101-33442300 | 1.902858 | NPC1  RMC1 | 1-16/25  5-19/19 | Membrane raft organization  Regulation of autophagy |
| chr25:19015900-19035000 | 0.268511 | CRYM-AS1 | 3/3 | Not available |
| chr25:34497300-34500900 | 1.7447 | OR4C15-like2 | 2/2 | Signal transduction |
| chr26:35121300-35126400 | 1.63559 | CYP2C31 | 2-3/9 | Drug metabolism |
| chr27:8383100-8398000 | 1.97891 | POLB | 12-13/13 | DNA repair, base-excision repair |
| chr27:10956200-10971400 | 2.0154 | ADAM18 | 18-20/20 | Proteolysis |

Read depth>1.5; duplication in Nubian ibex. Read depth<0.8; duplication in reference genome.
